# Fluorescent markers for the Spitzenkörper and exocytosis in *Zymoseptoria tritici*^☆^

**DOI:** 10.1016/j.fgb.2015.04.014

**Published:** 2015-06

**Authors:** M. Guo, S. Kilaru, M. Schuster, M. Latz, G. Steinberg

**Affiliations:** School of Biosciences, University of Exeter, Exeter EX4 4QD, UK

**Keywords:** ZtGFP, *Z. tritici* codon-optimised green fluorescent protein, Zt, *Z. tritici*, Sec4, GTPase, Mlc1, myosin light chain, Exo70, subunit of the exocyst complex, Spa2, polarity protein, *sdi1*, succinate dehydrogenase 1, MT, Microtubule, tub2, α tubulin, RB and LB, right and left border, Exocytosis, Secretion, Hyphal tip, Pathogenic fungi, Septoria tritici blotch, *Mycosphaerella graminicola*

## Abstract

Fungal hyphae are highly polarized cells that invade their substrate by tip growth. In plant pathogenic fungi, hyphal growth is essential for host invasion. This makes polarity factors and secretion regulators potential new targets for novel fungicides. Polarization requires delivery of secretory vesicles to the apical Spitzenkörper, followed by polarized exocytosis at the expanding cell tip. Here, we introduce fluorescent markers to visualize the apical Spitzenkörper and the apical site of exocytosis in hyphae of the wheat pathogen *Zymoseptoria tritici*. We fused green fluorescent protein to the small GTPase ZtSec4, the myosin light chain ZtMlc1 and the small GTPase ZtRab11 and co-localize the fusion proteins with the dye FM4-64 in the hyphal apex, suggesting that the markers label the hyphal Spitzenkörper in *Z. tritici*. In addition, we localize GFP-fusions to the exocyst protein ZtExo70, the polarisome protein ZtSpa2. Consistent with results in the ascomycete *Neurospora crassa*, these markers did localize near the plasma membrane at the hyphal tip and only partially co-localize with FM4-64. Thus, these fluorescent markers are useful molecular tools that allow phenotypic analysis of mutants in *Z. tritici*. These tools will help develop new avenues of research in our quest to control STB infection in wheat.

## Highlights

• We establish *Z. tritici* polarity markers ZtSec4, ZtMlc1, ZtRab11, ZtExo70 and ZtSpa2.
• All markers localize correctly, labeling the Spitzenkörper and sites of polar exocytosis.
• We provide 5 carboxin-resistance conveying vectors for integration of all markers into the *sdi1* locus.
• We provide 5 hygromycin B-resistance conveying vectors for random integration of all markers.

## 1 Introduction

Fungal hyphae grow by polar extension of their tip. Indeed, tip growth underlies invasive pathogen growth (Steinberg, 2007). Continuous polarized tip growth requires the constant delivery of “supplies” to the expanding cell apex. This involves the tip-wards transport of secretory vesicles. These Golgi-derived membranous structures were considered carriers that contain proteins for apical exocytosis (Pantazopoulou et al., 2014). Vesicle-mediated secretion begins with the formation of Golgi carriers at the trans-Golgi network. During delivery to the growing hyphal tip, Golgi-membranes mature into post-Golgi membranes that carry the small GTPase Rab11 (Pantazopoulou et al., 2014), and finally cluster in the apical Spitzenkörper (Sanchez-Leon et al., 2015; Pantazopoulou et al., 2014). This vesicle accumulation is found in ascomycetes and basidiomycetes and is thought to act as a “vesicle-supply center”, from where secretory vesicles are released for apical exocytosis (Bartnicki-Garcia et al., 1995, 1989). Consequently, the Spitzenkörper has a central role in directed growth of ascomycete filamentous fungi (Riquelme et al., 1998; Virag and Harris, 2006a; Riquelme and Sanchez-Leon, 2014). The Spitzenkörper can be labeled with the dye FM4-64 (Fischer-Parton et al., 2000; Virag and Harris, 2006b; Fischer et al., 2008; Jones and Sudbery, 2010; Sanchez-Leon et al., 2015), and co-localization studies with this dye and green-fluorescent protein fusions revealed numerous proteins that localize to this fungal-specific structure (overview in Riquelme and Sanchez-Leon, 2014). Amongst these are the GTPase Sec4 and Mlc1 (Sanchez-Leon et al., 2015; Giraldo et al., 2013; Jones and Sudbery, 2010). The exact role of these proteins in fungal growth is not known, but Mlc1 is a myosin light chain, implicated in controlling secretory motor class V myosin Myo2p in budding yeast (Stevens and Davis, 1998; Wagner et al., 2002). In *Aspergillus nidulans*, a myosin-V is involved in exocytosis (Taheri-Talesh et al., 2012) and localizes to the Spitzenkörper (Pantazopoulou et al., 2014). This suggests that Mlc1, together with myosin-V, is involved in actin-dependent steps in exocytosis. The small GTPase Sec4 is also implicated in protein secretion in *Candida albicans* and *Aspergillus niger* (Mao et al., 1999; Jones and Sudbery, 2010; Punt et al., 2001). The final step of fusion of secretory vesicles with the plasma membrane is thought to be supported by the exocyst. This multi-protein complex consists of eight proteins, including Exo70 (TerBush et al., 1996), and is considered to tether secretory vesicles to the plasma membrane prior to polarized exocytosis (Pfeffer, 1999; Guo et al., 2000; He and Guo, 2009). In filamentous fungi, the exocyst protein Exo70 localizes at the apex and only partially localizes with the FM4-64-positive Spitzenkörper (Riquelme et al., 2014; Sanchez-Leon et al., 2015) or Spitzenkörper-located proteins (Taheri-Talesh et al., 2008). Finally, polarized growth of fungi depends on a multi-protein complex, the polarisome (Harris et al., 2005). A central component of the polarisome is Spa2, which localizes to the hyphal apex in various fungi (Knechtle et al., 2003; Zheng et al., 2003; Virag and Harris, 2006b). In budding yeast, Spa2 is thought to be a scaffold protein that interacts with numerous polarity-determining proteins (Sheu et al., 1998; Van Drogen and Peter, 2002). It is not clear whether this role is conserved in filamentous fungi, but it was shown that Spa2 has essential roles in cell polarity and morphology in various model systems (Li et al., 2014; Meyer et al., 2008; Carbo and Perez-Martin, 2008; Virag and Harris, 2006b; Zheng et al., 2003).

In this study, we choose to establish homologues of Sec4, Mlc1, Rab11, Spa2, Exo70 as secretion and polarity markers in *Zymoseptoria tritici.* We generated fusion proteins of these proteins and a green-fluorescent protein, codon-optimized for use in the wheat pathogen *Z. tritici* (ZtGFP, more details in Kilaru et al., 2015c). We show that all markers localize within growing hyphae and concentrate at distinct regions in the hyphal apex. Thus, these 5 marker proteins are useful tools to investigate the molecular mechanism of polarized tip growth in the wheat pathogen *Z. tritici*.

## 2 Materials and methods

### 2.1 Bacterial and fungal strains and growth conditions

*Escherichia coli* strain DH5α was used for the maintenance of plasmids. *Agrobacterium tumefaciens* strain EHA105 (Hood et al., 1993) was used for maintenance of plasmids and subsequently for *A. tumefaciens*-mediated transformation of *Z. tritici. E. coli* and *A. tumefaciens* were grown in DYT media (tryptone, 16 g/l; yeast extract, 10 g/l; NaCl, 5 g/l; with 20 g/l agar added for preparing the plates) at 37 °C and 28 °C respectively. The fully sequenced *Z. tritici* wild-type isolate IPO323 (Goodwin et al., 2011; Kema and van Silfhout, 1997) was used as recipient strain for the genetic transformation experiments. The isolate was inoculated from stocks stored in NSY glycerol (nutrient broth, 8 g/l; yeast extract, 1 g/l; sucrose, 5 g/l; glycerol, 700 ml/l), at −80 °C onto solid YPD agar (yeast extract, 10 g/l; peptone, 20 g/l; glucose, 20 g/l; agar, 20 g/l) and grown at 18 °C for 4–5 days.

### 2.2 Identification of *Z. tritici* homologues and bioinformatics

To identify homologues of the chosen marker proteins, we screened the published sequence of *Z. tritici* (http://genome.jgi.doe.gov/Mycgr3/Mycgr3.home.html) using protein BLAST (http://blast.ncbi.nlm.nih.gov/Blast.cgi) and the *Ustilago maydis* protein sequence of Rab11 (NCBI accession number: XP_757798.1), *C. albicans* protein sequence of Sec4 (NCBI accession number: KGR01655.1; Bishop et al., 2010) and the *Magnaporthe oryzae* proteins Exo70*,* Mlc1 and Spa2 (NCBI accession numbers: XP_003714759.1, XP_007284752.1 and XP_003716178.1, respectively; Giraldo et al., 2013). Sequences were obtained from the NCBI server (http://www.ncbi.nlm.nih.gov/pubmed) and comparison was done using CLUSTAL W (http://www.ebi.ac.uk/Tools/msa/clustalw2/) and EMBOSS Needle (http://www.ebi.ac.uk/Tools/psa/emboss_needle/). The start codon of the open reading frame was predicted by the annotation in the JIG data base (http://genome.jgi.doe.gov/Mycgr3/Mycgr3.home.html) and confirmed by sequence comparison with other fungal homologues. Domain structures were analyzed in PFAM (http://pfam.xfam.org/search/sequence). Finally, phylogenetic trees were generated in MEGA5.2, using a Maximum Liklihood method, followed by 1000 bootstrap cycles (http://www.megasoftware.net/; Tamura et al., 2011).

### 2.3 Molecular cloning

All the vectors used in this study were generated by *in vivo* recombination in the yeast *Saccharomyces cerevisiae* DS94 (MATα, *ura3-52*, *trp1-1*, *leu2-3*, *his3-111*, and *lys2-801* (Tang et al., 1996) following published procedures (Raymond et al., 1999; Kilaru and Steinberg, 2015). For all the recombination events, the fragments were amplified with 30 bp homologous sequences to the upstream and downstream of the fragments to be cloned (see Table 1 for primer details). PCR reactions and other molecular techniques followed standard protocols (Sambrook and Russell, 2001). All restriction enzymes and reagents were obtained from New England Biolabs Inc (NEB, Herts, UK).

### 2.4 Targeted ectopic integration vectors to visualize polarity factors

The vector pCZtGFPSec4 contains *ztgfp* (Kilaru et al., 2015c) fused to full-length *ztsec4* under the control of *zttub2* promoter and terminator sequences for integration into the *sdi1* locus of *Z. tritici* by using carboxin as selection agent. A 13,603 bp fragment of pCZtGFPTub2 (digested with *Xho*I*,* unpublished vector), 717 bp *ztgfp* (amplified with SK-Sep-101 and MG-178; Table 2), 897 bp full-length *ztsec4* gene (amplified with MG-179 and MG-180; Table 2) were recombined in yeast *S. cerevisiae* to obtain the vector pCZtGFPSec4 (Fig. 2A).

The vectors pCZtGFPExo70, pCZtGFPMlc1 and pCZtGFPSpa2 contains *ztgfp* fused to full-length *ztexo70*, *ztmlc1* and *ztspa2* under the control of *zttub2* promoter and terminator sequences for integration into the *sdi1* locus of *Z. tritici* by using carboxin as selection agent. A 13,603 bp fragment of pCZtGFPTub2 (digested with *Xho*I*,* unpublished vector), 717 bp *ztgfp* (amplified with SK-Sep-101 and MG-174; Table 2) and either 1890 bp full-length *ztexo70* gene (amplified with MG-183 and MG-184; Table 2), 984 bp full-length *ztmlc1* gene (amplified with MG-185 and MG-186; Table 2) or 2757 bp full-length *ztspa2* gene (amplified as two fragments with MG-189 and MG-191; MG-192 and MG-190; Table 2) were recombined in yeast *S. cerevisiae* to obtain the vectors pCZtGFPExo70, pCZtGFPMlc1 and pCZtGFPSpa2 respectively (Fig. 2A).

The vector pCeGFPRab11 contains *egfp* fused to the full-length *ztrab11* under the control of constitutive *Zttub2* promoter and terminator sequences for targeted integration into the *sdi1* locus of *Z. tritici* by using carboxin as selection agent. A 14,907 bp fragment of pCeGFPTub2 (Schuster et al., 2015; digested with *Xho*I) and 807 bp full-length z*trab11* gene (amplified with SK-Sep-65 and SK-Sep-66; Table 2) were recombined in yeast *S. cerevisiae* to obtain the vector pCeGFPRab11 (Fig. 2A).

### 2.5 Random ectopic integration vectors to visualize polarity factors

The vectors pHZtGFPSec4, pHZtGFPExo70, pHZtGFPMlc1 and pHZtGFPSpa2 contain *ztgfp* (Kilaru et al., 2015c) fused to the full-length *ztsec4, ztexo70, ztmlc1 and ztspa2* under the control of *zttub2* promoter and terminator sequences for random ectopic integration into the genome of *Z. tritici* using hygromycin B as selection agent. A 14,428 bp fragment of pCZtGFPSec4 (digested with *Bam*HI and *Bgl*II), a 15,730 bp fragment of pCZtGFPExo70 (digested with *Bam*HI), a 14,515 bp fragment of pCZtGFPMlc1 (digested with *Bam*HI and *Bgl*II), a 16,597 bp fragment of pCZtGFPSpa2 (digested with *Bgl*II) were individually recombined with 1806 bp hygromycin resistance cassette (amplified with MG-181 and MG-182; Table 2) in yeast *S. cerevisiae* to obtain the vectors pHZtGFPSec4, pHZtGFPExo70, pHZtGFPMlc1 and pHZtGFPSpa2 respectively (Fig. 2D). Note that these vectors were derived from carboxin resistance conferring vectors pCZtGFPSec4, pCZtGFPExo70, pCZtGFPMlc1 and pCZtGFPSpa2 (Fig. 2A) and as such it contain part of the succinate dehydrogenase gene, carrying the mutation H267L and succinate dehydrogenase terminator. However, these fragments are of no significance.

The vector pHeGFPRab11 contains *egfp* fused to the full-length *ztrab11* under the control of constitutive *zttub2* promoter and terminator sequences for ectopic random integration by using hygromycin B as selection agent. A 14,647 bp fragment of pCeGFPRab11 (Fig. 2A; digested with *Bam*HI) and 1510 hygromycin resistance cassette (amplified with SK-Sep-128 and SK-Sep-129; Table 2) were recombined in yeast *S. cerevisiae* to obtain the vector pHeGFPRab11 (Fig. 2D). Note that this vector was derived from carboxin resistance conferring vector pCeGFPRab11 (Fig. 2A) and as such it contains part of the succinate dehydrogenase gene, carrying the mutation H267L and succinate dehydrogenase terminator. However, these fragments are of no significance. Further details on vector construction and yeast recombination-based cloning is provided in (Kilaru and Steinberg, 2015).

### 2.6 *Z. tritici* transformation and molecular analysis of transformants

The vectors pCZtGFPSec4, pCZtGFPExo70, pCZtGFPMlc1, pCZtGFPSpa2 and pHeGFPRab11 were transformed into *A. tumefaciens* strain EHA105 by heat shock method (Holsters et al., 1978). *A. tumefaciens* mediated transformation *of Z. tritici* was performed as described previously by (Zwiers and De Waard, 2001) with the slight modifications. To confirm the integration of vector into the *sdi1* locus of *Z. tritici* and also to determine the copy number, Southern blot hybridizations were performed by using the standard procedures (Sambrook and Russell, 2001). 3 μg of genomic DNA of IPO323 and transformants obtained with various vectors were digested with *Bgl*II and separated on a 1.0% agarose gel and capillary transferred to a Hybond N^+^membrane (Amersham Pharmacia Biotech). 1014 bp *sdi1* probe (3′ end of the *sdi1^R^* gene and *sdi1* terminator) was generated using primers SK-Sep-10 and SK-Sep-13 (Table 2) using DIG labeling PCR mix (Life Science Technologies, Paisley, UK). Hybridizations were performed at 62 °C for overnight and autoradiographs were developed after an appropriate time period.

### 2.7 Plate growth assay

YPD agar was used to examine plate growth of IPO323, IPO323_CZtGFPSec4, IPO323_CZtGFPExo70, IPO323_CZtGFPMlc1, IPO323_CZtGFPSpa2 and IPO323_HeGFPRab11. For better visualization of the colonies, 1% activated charcoal was added to the media. *Z. tritici* cells were grown on YPD agar for 5 days at 18 °C. The cell density was adjusted to an optical density of 0.4 at 660 nm in sterile water. The cell cultures were serially diluted (10 times each) in sterile water. The serial diluted cultures were then applied as 5 μl droplets on YPD agar with 1% charcoal and grown at 18 °C for 5 days. Photographs of the relative colony densities were taken using a canon digital IXUS 80 IS camera (Canon, Surrey, UK).

### 2.8 Epi-fluorescence microscopy

Fluorescence microscopy was performed as previously described (Kilaru et al., 2015b). In brief, the cells were inoculated in YG media and grown at 24 °C with 100 rpm for 24 h and placed onto a 2% agar cushion for direct observation using a motorized inverted microscope (IX81; Olympus, Hamburg, Germany), equipped with a PlanApo 100×/1.45 Oil TIRF (Olympus, Hamburg, Germany). Fluorescent tags and dyes were exited using a VS-LMS4 Laser Merge System with solid-state lasers (488 nm/50 mW or 75 mW and 561 nm/50 mW or 75 mW; Visitron Systems, Puchheim, Germany) and single images or *z*-Stacks, using an objective piezo (Piezosystem Jena GmbH, Jena, Germany), over 6 μm depth with a *z* resolution of 0.2 μm were captured with 150 ms exposure. In addition a DIC image was taken for each cell using a CoolSNAP HQ2 camera (Photometrics/Roper Scientific, Tucson, USA). Overlays of the fluorescent and DIC images as well as the pseudo-colored images were generated using MetaMorph (Molecular Devices, Wokingham, UK). All parts of the system were under the control of the software package MetaMorph (Molecular Devices, Wokingham, UK).

### 2.9 FM4-64 staining

The dye FM4-64 (Molecular Probes/Invitrogen, Paisley, UK) was used to label the apical Spitzenkörper (Fischer-Parton et al., 2000). Cells of strains IPO323_CZtGFPMlc1, IPO323_CZtGFPSec4, IPO323_HeGFPRab11, and IPO323_CZtGFPExo70 were inoculated in YG media and grown at 24 °C with 100 rpm for 24 h. 1 ml of those cultures was incubated in YG media containing 1 μM FM4-64 for 10 min at 24 °C, shaking at 100 rpm. The cells were washed by centrifugation for 5 min at 5,000 rpm and re-suspended in fresh YG media and incubated at 24 °C with 100 rpm for additional 15 min followed by observed using a dual-line beam splitter (Dual-View 2 Multichannel Imaging System; Photometrics, Tucson, USA). Z-Stacks over 6 μm depth with a z resolution of 0.2 μm and an exposure time of 150 ms were taken. The green fluorescent labeled polarity markers were excited using the 488 nm laser at 20–100% output power and the FM4-64 dye was excited using the 561 nm laser at 50% output power. In addition a bright field image was taken for each cell to get the blue outline in the overlaid images.

## 3 Results and discussion

### 3.1 Identification of ZtSec4, ZtExo70, ZtMlc1, ZtSpa2 and ZtRab11

**Fig. 1:**
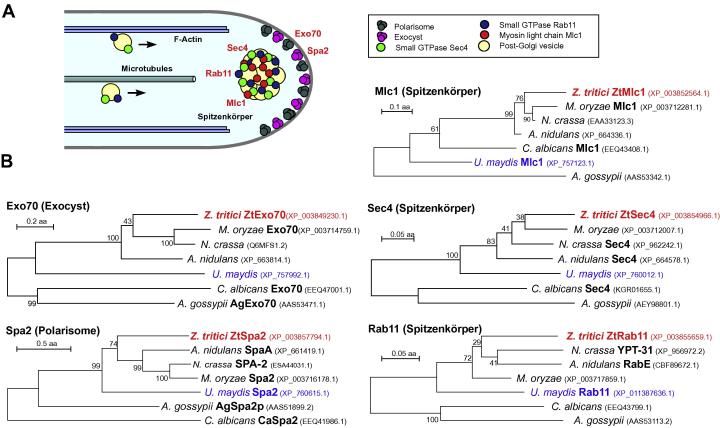
Cell polarity markers in *Z. tritici.* (A) Schematic drawing of a growing hyphal tip of a filamentous fungus. Post-Golgi vesicles are delivered by the cytoskeleton to the hyphal tip. In many fungi, they accumulate in the apical Spitzenkörper. The myosin light chain Mlc1 and the small GTPases Sec4 and Rab11/Ypt-31 concentrate in the Spitzenkörper. The exocyst complex is thought to support tethering of secretory vesicles at the plasma membrane and localizes in an apical cap in most fungi. A similar localization is described for the polarisome protein Spa2. The diagram is based on published data in several fungi, including *N. crassa* (Sanchez-Leon et al., 2015; Araujo-Palomares et al., 2009), *A. nidulans* (Virag and Harris, 2006b; Pantazopoulou et al., 2014), *M. oryzae* (Giraldo et al., 2013), *C. albicans* (Jones and Sudbery, 2010), *A. gossypii* (Köhli et al., 2008), *U. maydis* (Carbo and Perez-Martin, 2008), *A. niger* (Meyer et al., 2008). Note that Exo70 in *N. crassa* does not form an apical cap, but localizes to the Spitzenkörper (Sanchez-Leon et al., 2015). The same localization was found in *A. gossypii* fast-growing hyphae (Köhli et al., 2008). In these cells, Spa2 is also concentrated in the Spitzenkörper (Köhli et al., 2008). This corresponds with a partial co-localization of Spa2 and the Spitzenkörper in *N. crassa* and *A. nidulans* was reported (Lichius et al., 2012; Virag and Harris, 2006a,b). (B) Phylogenetic trees comparing the predicted full-length amino acid sequence of fungal homologues of the exocyst protein Exo70, the polarisome protein Spa2, the vesicle associated GTPase Sec4, the myosin-light chain Mlc1 and the GTPase Rab11/Ypt31. The *Z. triti*ci orthologues, used in this study, are indicated in bold and red. The basidiomycete *U. maydis* is indicated in blue. Where available, published protein names are provided in bold. NCBI accession numbers are given behind species names (http://www.ncbi.nlm.nih.gov/pubmed). Maximum likelihood trees were generated using MEGA5.2 (Tamura et al., 2011). Bootstrap values are indicated at branching points.

As a first step toward establishing polarity markers, we screened the *Z. tritici* published genome sequence of IPO323 at the Joint Genome Institute (http://genome.jgi.doe.gov/Mycgr3/Mycgr3.home.html), using protein sequences of Rab11 from *U. maydis* (Fuchs and Steinberg, 2005), Sec4 from *C. albicans* (Bishop et al., 2010) and Exo70*,* Mlc1 and Spa2 from *M. oryzae* (Giraldo et al., 2013; see materials and methods for more detail). In this way, we identified homologues of these polar localized proteins in *Z. tritici* (ZtRab11: JGI protein number: 28304, NCBI accession number: XP_003855659.1), ZtSec4 (JGI protein number: 99145; NCBI accession number: XP_003854966.1), ZtExo70 (JGI protein number: 101338; NCBI accession number: XP_003849230.1), ZtMlc1 (JGI protein number: NCBI 104579; accession number: XP_003852564.1) and ZtSpa2 (JGI protein number: 106740; NCBI accession number: XP_003857794.1). The start and the stop codons of each open reading frame were confirmed by comparison with homologous proteins. We next compared the predicted amino acid sequences of these *Z. tritici* proteins with sequences of orthologues in the ascomycetes *M. oryzae*, *Neurospora crassa*, *A. nidulans*, *Ashbya. gossypii*, *C. albicans* and the basidiomycetes *U. maydis*, using a maximum likelihood approach, provided by the Molecular Evolutionary Genetics Analysis software MEGA 5.2 (Tamura et al., 2011; see materials and methods). The maximum likelihood method is a well-established way of inferring phylogenetic trees from DNA (Felsenstein, 1981) or protein sequences (Kishino et al., 1990). All predicted *Z. tritici* proteins grouped within their orthologues sequences from ascomycete filamentous fungi (Fig. 1B; Table 1). In addition, all *Z. tritici* shared similar domain structures with selected orthologues (Table 1). Interestingly, this analysis reveals that all chosen polarity marker proteins in filamentous ascomycetes are more similar to the basidiomycete *U. maydis* than to the ascomycete fungi *A. gossypii* and *C. albicans* (Fig. 1B).

### 3.2 Vectors for targeted ectopic integration of constructs with GFP fused polarity factors

**Fig. 2:**
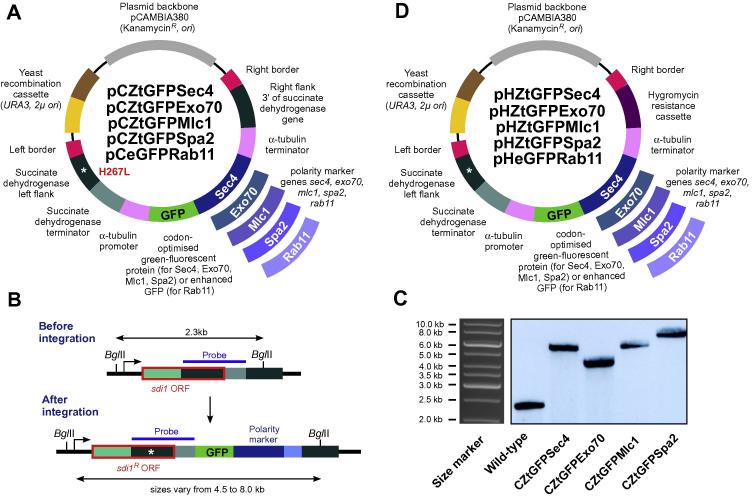
Vectors for integration of polarity markers into the *sdi1* locus of *Z. tritici*. (A) All vectors carry N-terminal fusions of either codon-optimized eGFP (ZtGFP; Kilaru et al., 2015c) or enhanced GFP (eGFP) and polarity markers (for explanation see Fig. 1A and main text). All vectors contain the H267L point mutation in a stretch of *sdi1* sequence, which allows targeted integration into the *sdi1* locus of *Z. tritici*, thereby conferring resistance to the fungicide carboxin (for more information see carboxin paper, this issue). Note that fragments are not drawn to scale. For more accurate information on fragment sizes see main text. (B) Diagram showing the organization of the *sdi1* locus before and after integration of the GFP-encoding vectors. Note that integration of the point mutated *sdi1* left flank (see (A); point mutation indicated by asterisk) replaces a part of the *sdi1* open reading frame (*sdi1* ORF) and confers carboxin resistance (*sdi1^R^* ORF). Successful integration of the vector increases the size of a DNA fragment after digestion with the restriction enzyme *Bgl*II and subsequent detection with a labeled DNA probe (blue bar). (C) Southern blots showing integration of pCZtGFPSec4, pCZtGFPExo70, pCZtGFPMlc1 and pCZtGFPSpa2 into the *sdi1* locus of *Z. tritici* IPO323. The blot was hybridised with *sdi1* probe. After digestion of the genomic DNA with *Bgl*II and subsequent hybridization with a DIG labeled DNA probe, shifts in the DNA fragment from 2.3 kb to 6.2 kb, 4.5 kb, 6.2 kb and 8.0 kb is detected. The size markers in the corresponding agarose gel are shown to the left. (D) Vectors for random integration of polarity marker constructs into the genome of *Z. tritici*. All vectors carry N-terminal fusions of either *Z. tritici* codon-optimized eGFP (for more information on ZtGFP; Kilaru et al., 2015c) or enhanced GFP (eGFP) and polarity markers (for explanation see Fig. 1A and main text). They are designed for random integration into the genome and confer hygromycin resistance. Note that these vectors were derived from carboxin resistance conferring vectors (A). As such they contain part of the succinate dehydrogenase gene, carrying the mutation H267L and succinate dehydrogenase terminator. However, these fragments are of no significance.

In order to visualize polarity factors, we constructed five different vectors pCZtGFPSec4, pCZtGFPExo70, pCZtGFPMlc1, pCZtGFPSpa2 and pCeGFPRab11 designed for targeted ectopic integration into *sdi1* locus of *Z. tritici* (for details on this locus Kilaru et al., 2015a). All five vectors carry a mutated downstream stretch of the *sdi1* sequence, carrying a carboxin resistance-conferring point mutation (H267L; Fig. 2A, left flank), and a sequence stretch downstream of *sdi1* (Fig. 2A, right flank of *sdi1*). Incorporation by homologous recombination mutates the *sdi1* gene and integrates the ZtGFP-marker fusion constructs into the *sdi1* locus (Fig. 2B; for details see Kilaru et al., 2015a). This results in a single integration of each construct without affecting other *Z. tritici* genes. Each vector carried either codon-optimised enhanced green fluorescent protein (ZtGFP, see Kilaru et al., 2015c) or enhanced green fluorescent protein (eGFP) fused to the N-terminal end of ZtSec4, ZtExo70, ZtMlc1, ZtSpa2 and ZtRab11. Expression of these fluorescent fusion proteins was driven by the α-tubulin promoter (for more information on the α-tubulin gene *tub2* see Schuster et al., 2015). All five vectors were built on the *Agrobacterium* binary vector pCAMBIA0380 (CAMBIA, Canberra, Australia). These vectors allow *A. tumefaciens*-based transformation into *Z. tritici*, which is based on the 25 bp imperfect directional repeat sequences of the T-DNA borders (right and left border, RB and LB; Fig. 2A). These vectors carry a kanamycin resistance gene, origins of replication for amplification in *E. coli* and *A. tumefaciens*. In addition, all the vectors carry a “yeast recombination cassette”, consisting of URA3 and 2μ *ori*, which enables yeast recombination-based cloning (for more details see Kilaru and Steinberg, 2015). Further details on the vectors can be found in the material and methods.

### 3.3 Vectors for random ectopic integration of constructs with GFP fused secretory components

We also constructed a further set of vectors pHZtGFPSec4, pHZtGFPExo70, pHZtGFPMlc1, pHZtGFPSpa2 and pHeGFPRab11, designed for random ectopic integration of the GFP-marker fusion proteins (Fig. 2D). These vectors were derived from the vectors described above and share most features, including *A. tumefaciens* – mediated transformation capacity and the capability to be used in yeast recombination-based cloning. In contrast, these vectors carry a hygromycin resistance conferring cassette, which allows transformation into strains that already contain another marker integrated in the *sdi1* locus. It needs to be noted that all vectors contain the *sdi1* downstream sequence (*sdi1* left flank and terminator). This sequence is a remnant of the cloning procedure and of no functional significance.

### 3.4 *Z. tritici* strains containing fluorescently labeled polarity markers

**Fig. 3:**
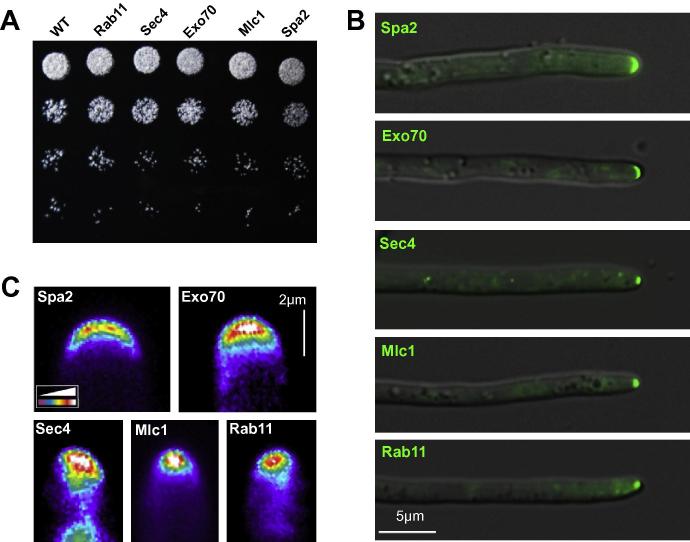
Localization of polarity markers in hyphal tips of *Z. tritici*. (A) Plate growth assay of IPO323 (WT), IPO323_HeGFPRab11 (Rab11), IPO323_CZtGFPSec4 (Sec4), IPO323_CZtGFPExo70 (Exo70), IPO323_CZtGFPMlc1 (Mlc1) and IPO323_CZtGFPSpa2 (Spa2). Colonies were grown for 5 days. No difference was found between all strains, suggesting that expression of the fluorescent markers is not affecting growth on solid media. (B) Localization of all florescent markers in hyphae of strains IPO323_CZtGFPSpa2 (Spa2), IPO323_CZtGFPExo70 (Exo70), IPO323_CZtGFPSec4 (Sec4), IPO323_CZtGFPMlc1 (Mlc1), and IPO323_HeGFPRab11 (Rab11). Fluorescent signals and corresponding DIC images were overlaid. Bar represents 5 micrometers. (C) Pseudo-color images of fluorescent signals off all marker proteins in hyphal tips. Note that Sec4, Mlc1 and Rab11 are focused in a single spot, whereas Spa2 and Exo70 form an apical cap. Signal intensities are provided in a color code (white = very strong signal, purple = weak signal). Bar represents 2 micrometers.

We next set out to visualize the localization of all 5 fluorescent marker proteins in *Z. tritici*. To this end, we transformed vectors pCZtGFPSec4, pCZtGFPExo70, pCZtGFPMlc1, pCZtGFPSpa2 and pHeGFPRab11 into *Z. tritici* strain IPO323, tested microscopically for GFP fluorescence and confirmed integration into the *sdi1* locus by Southern blotting. The genomic DNA was digested with *Bgl*II and hybridized with a *sdi1* probe (see Fig. 2C for localization of probe). In all cases, we found a single band at the expected size (CZtGFPSec4: 6.2 kb, CZtGFPExo70: 4.5 kb, CZtGFPMlc1: 6.2 kb and CZtGFPSpa2: 8.0 kb; Fig. 2B and C), confirming that all 4 fusion constructs were integrated into the *sdi1* locus as single copies. The resultant strains were named as IPO323_CZtGFPSec4, IPO323_CZtGFPExo70, IPO323_CZtGFPMlc1 and IPO323_CZtGFPSpa2, respectively. The vector pHeGFPRab11 was designed for the random ectopic integration into the genome. Consequently, integration of this vector into the resultant strain IPO323_HeGFPRab11 was not tested by Southern blotting. None of these strains showed growth defects on agar plates (Fig. 3A), suggesting that expression of the GFP-fusion proteins is not harmful to the cells.

### 3.5 Localization of the fluorescent markers in hyphae of *Z. tritici*

**Fig. 4:**
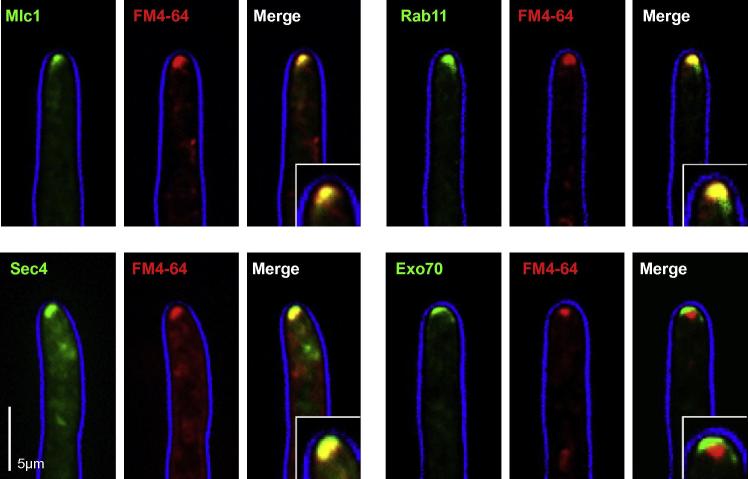
Co-visualization of the Spitzenkörper and polarity markers in hyphae of *Z. tritici.* Hyphae of strains IPO323_CZtGFPMlc1, IPO323_CZtGFPSec4, IPO323_HeGFPRab11, and IPO323_CZtGFPExo70 were stained with FM4-64 (indicated in red), which is labeling the Spitzenkörper in other filamentous ascomycetes (Fischer-Parton et al., 2000). The myosin light chain Mlc1 (Mlc1, green), the small GTPases Sec4 (Sec4, green) and Rab11 (Rab11, green) are co-localizing with the FM4-64 signal at the tip of the hyphal cell. This localization confirms results in other ascomycete fungi (Sanchez-Leon et al., 2015; Pantazopoulou et al. 2014; Giraldo et al., 2013; Jones and Sudbery, 2010). The exocyst protein Exo70 does only partially co-localize with the Spitzenkörper (see merged image, lower right, Exo70 in green, FM4-64 in red). This is in agreement with a role of the exocyst in tethering secretory vesicles to the plasma membrane and confirms localization data in *N. crassa* and *Candida albicans* (Riquelme et al., 2014; Jones and Sudbery, 2010). Note that the cell edge is provided in blue. The bar represents 5 micrometers.

We next investigated the localization of all ZtGFP-marker fusion proteins. In hyphae of the *Z. tritici* strains, all GFP-marker proteins localize to the growth region at the cell tip (Fig. 3B). We found that ZtSec4, ZtMlc1 and ZtRab11 was concentrating in a dot-like signal in cell apex (Fig. 3C, lower panels), which is in agreement with previous reports in other ascomycetes (Sanchez-Leon et al., 2015; Pantazopoulou et al., 2014; Giraldo et al., 2013; Jones and Sudbery, 2010; Crampin et al., 2005). In other fungi, Sec4, Mlc1 and Rab11 have been shown to co-localize with the Spitzenkörper, which was stained by the dye FM4-64 (Sanchez-Leon et al., 2015; Pantazopoulou et al., 2014; Giraldo et al., 2013; Jones and Sudbery, 2010). We tested if the dot-like accumulation of the *Z. tritici* marker proteins also co-localizes with the Spitzenkörper by co-visualization of FM4-64 and GFP fluorescence. Indeed, we found that the strong fluorescent signal of GFP-ZtSec4, GFP-ZtMlc1 and GFP-ZtRab11 co-localized with the marker dye FM4-64 (Fig. 4). However, we do not see different sub-localization of markers within the Spitzenkörper, as described in *N. crassa* (Sanchez-Leon et al., 2015). This may be due to the very small size of this structure in *Z. tritici* (0.56 ± 0.43 μm, n = 10), compared to the large Spitzenkörper in *N. crassa* (∼3 × 2.5 μm, dimensions taken from Fig. 1 in Fajardo-Somera et al., 2015). In addition, it is important to note that some variations in these localization patterns occurred. This was most obvious for Sec4, which in some hyphae was distributed very diffusely. Presently, it is not clear whether this is due to the ectopic expression of GFP-Sec4 in a wildtype background, or whether it reflects a variation in the growth rate of individual hyphae, which did affect localization of polar proteins in *A. gossypii* (Köhli et al., 2008). However, we conclude that all three marker proteins stain the Spitzenkörper in hyphae of *Z. tritici*.

The exocyst component ZtExo70 and the polarisome protein ZtSpa2 localized in an apical crescent (Figs. 3C and 4). This localization is in agreement with their localization in hyphal tips of *C. albicans* (Crampin et al., 2005; Jones and Sudbery, 2010), *A. gossypii* (Köhli et al., 2008) and *N. crassa*, where both proteins only partially co-localize with the Spitzenkörper (Araujo-Palomares et al., 2009; Riquelme et al., 2014). Unfortunately, GFP-ZtSpa2-containing hyphae did not form a distinct FM4-64 stainable Spitzenkörper, suggesting that the expression of this polarisome component is affecting the growth rate of the hyphae. However, the location of ZtExo70 in agreement with the proposed function of the exocytst as a tethering complex for secretory vesicles (He and Guo, 2009).

## 4 Conclusion

*Z. tritici* infects plant tissue by invasive hyphal growth (for review see Steinberg, 2015), which most likely depends on polarized secretion of secretory vesicles and their content at the expanding hyphal tip. In this study we established 5 markers for polarized secretion. Fluorescent versions of ZtSec4 and ZtMlc1 are located at the Spitzenkörper, although Sec4 is less focused and may label additional secretory vesicles. ZtExo70 and ZtSpa2 mark the region of polarized exocytosis, where the polarisome complex supports actin-filament elongation. Using these markers in combination with mutant phenotypes will help understanding the molecular basis of polarized secretion. This holds the promise of new vistas of research in the development of novel fungicides to control this important wheat pathogen.

## Figures and Tables

**Table 1:** Bioinformatics of putative *Z. tritici* polarity marker proteins.

	Length^a^		Domains^b^		Identity^c^	Reference^d^
Mlc1	*Z. tritici*	*M. oryzae*	*Z. tritici*	*M. oryzae*	72.8%	Giraldo et al. (2013)
	140	150	EF-hand (3.1e−05)	–		
			EF-hand (6.4e−09)	EF-hand (1.8e−08)		

Exo70	*Z. tritici*	*M. oryzae*	*Z. tritici*	*M. oryzae*	42.3%	Giraldo et al (2013)
	630	632	Exo70 (4.9e−68)	Exo70 (2.9e−66)		

Spa2	*Z. tritici*	*A. nidulans*	*Z. tritici*	*A. nidulans*	31.0%	Virag and Harris (2006b)
	918	906	Spa2-GIT (2.4e−13)	Spa2-GIT (1.7e−13)		
			Spa2-GIT (3.8e−07)	Spa2-GIT (2.1e−08)		

Sec4	*Z. tritici*	*M. oryzae*	*Z. tritici*	*M. oryzae*	84.7%	Giraldo et al. (2013)
	207	206	Ras (3.4e−65)	Ras (2.5e−64)		

Rab11	*Z. tritici*	*A. nidulans*	*Z. tritici*	*A. nidulans*	78.9%	Pantazopoulou et al. (2014)
	211	210	Ras (1.6e−62)	Ras (5.5e−63)		

a Given in amino acids.

b Determined in PFAM (http://pfam.xfam.org/search/sequence) with error probability in brackets.

c Determined in EMBOSS Needle (http://www.ebi.ac.uk/Tools/psa/emboss_needle/).

d Reference for comparison.

**Table 2:** Primers used in this study.

Primer name	Direction	Sequence (5′ to 3′)^a^
MG-174	Antisense	TTGTAGAGCTCGTCCATGCCG
MG-178	Antisense	*AAGAAAGTCATAATTCCGACTGCCGGCCAT*CTTGTAGAGCTCGTCCATGCCG
MG-179	Sense	ATGGCCGGCAGTCGGAATTATG
MG-180	Antisense	*CCACAAGATCCTGTCCTCGTCCGTCGTCGC*TTAACAGCAGTTCTTCCCAAGT
MG-181	Sense	*CTCTCATAAGAGCTTGGCTGTCGACTCCTC*CCCAACTGATATTGAAGGAGCA
MG-182	Antisense	*TAAACGCTCTTTTCTCTTAGGTTTACCCGC*CCCGATCTAGTAACATAGATGA
MG-183	Sense	*ATCACCCTCGGCATGGACGAGCTCTACAAG*ATGGTGGGCGCAAGGCATGCCG
MG-184	Antisense	*CCACAAGATCCTGTCCTCGTCCGTCGTCGC*TCAACCCAGAGACGCCAGAATA
MG-185	Sense	*ATCACCCTCGGCATGGACGAGCTCTACAAG*ATGGTACGTCCCCCCCCGTGCG
MG-186	Antisense	*CCACAAGATCCTGTCCTCGTCCGTCGTCGC*TCAGTTCTGCAAAATCATCTTG
MG-189	Sense	*ATCACCCTCGGCATGGACGAGCTCTACAAG*ATGTCCATGTCACGTCTACCGC
MG-190	Antisense	*CCACAAGATCCTGTCCTCGTCCGTCGTCGC*CTACCGGTACGGATCATAGTCA
MG-191	Antisense	CGCAGCGTCATTTTGATTTGAC
MG-192	Sense	AAGAGTCGACCACCCAGGAAGT
SK-Sep-10	Sense	*TGGCAGGATATATTGTGGTGTAAACAAATT*GACCTTCCACATCTACCGATGG
SK-Sep-13	Sense	CTTCCGTCGATTTCGAGACAGC
SK-Sep-65	Sense	A*TCACTCTCGGCATGGACGAGCTGTACAAG*ATGGCGAACGACGAATACGATGT
SK-Sep-66	Antisense	*CCACAAGATCCTGTCCTCGTCCGTCGTCGC*TCAACAGCACTGTCCGCTCTTC
SK-Sep-101	Sense	*CATCACTCACATCCGCATACCACCATCGCC*ATGGTCTCCAAGGGCGAGGAG
SK-Sep-128	Sense	*CTCTCATAAGAGCTTGGCTGTCGACTCCTC*GAATTCGAGCTCGGTACCCAACT
SK-Sep-129	Antisense	*CTTTTCTCTTAGGTTTACCCGCGTTGAAGT*GCGTTAACACTAGTCAGATCTACC

a *Italics* indicate part of the primer that is complementary with another DNA fragment, to be ligated by homologous recombination in *S. cerevisiae*.
